# Residential Treatment for Combat-Related Posttraumatic Stress Disorder: Identifying Trajectories of Change and Predictors of Treatment Response

**DOI:** 10.1371/journal.pone.0101741

**Published:** 2014-07-24

**Authors:** Joseph M. Currier, Jason M. Holland, Kent D. Drescher

**Affiliations:** 1 Psychology Department, University of South Alabama, Mobile, Alabama, United States of America; 2 Department of Psychology, University of Nevada, Las Vegas, Las Vegas, Nevada, United States of America; 3 National Center for PTSD, Dissemination and Training Division, Menlo Park, California, United States of America; Univ of Toledo, United States of America

## Abstract

**Background:**

Combat-related posttraumatic stress disorder (PTSD) can be a difficult condition to treat and has been associated with serious medical and economic issues among U.S. military veterans. Distinguishing between treatment responders vs. non-responders in this population has become an important public health priority. This study was conducted to identify pre-treatment characteristics of U.S. veterans with combat-related PTSD that might contribute to favorable and unfavorable responses to high value treatments for this condition.

**Method:**

This study focused on 805 patients who completed a VHA PTSD residential program between 2000 and 2007. These patients completed the PTSD Clinical Checklist at pre-treatment, post-treatment, and a four-month follow-up assessment. Latent growth curve analysis (LCGA) was incorporated to determine trajectories of changes in PTSD across these assessments and whether several key clinical concerns for this population were associated with their treatment responses.

**Study Findings:**

LCGA indicated three distinct trajectories in PTSD outcomes and identified several clinical factors that were prospectively linked with changes in veterans' posttraumatic symptomatology. When compared to a group with high PTSD symptom severity that decreased over the program but relapsed at follow-up (41%), the near half (48.8%) of the sample with an improving trajectory had less combat exposure and superior physical/mental health. However, when compared to a minority (10.2%) with relatively low symptomatology that also remained somewhat stable, patients in the improving group were younger and also reported greater combat exposure, poorer physical/mental health status, and more problems with substance abuse before the start of treatment.

**Conclusions:**

Findings suggest that veterans are most likely to benefit from residential treatment in an intermediate range of symptoms and risk factors, including PTSD symptom severity, history of combat exposure, and comorbid issues with physical/mental health. Addressing these factors in an integrative manner could help to optimize the effectiveness of treatments of combat-related PTSD in many cases.

## Introduction

Treating combat-related posttraumatic stress disorder (PTSD) effectively is a public health priority. The costs of PTSD to society can be quite serious compared to other possible psychiatric conditions that may emerge following war-zone service [1]–[3]. Although most military veterans do not suffer from long-term consequences after deployment, an estimated 18.7% to 30% of Vietnam Veterans met criteria for PTSD at some point after returning to civilian life [4], [5]. Research with Iraq/Afghanistan Veterans has similarly documented PTSD prevalence rates of approximately 12% to 20% among those who served in combat operational capacities (e.g., infantry) [6], [7]. The number of new PTSD cases in the Veterans Health Administration (VHA) has accordingly more than doubled since the start of these new wars [8] and compensation for this condition has increased dramatically during this period among U.S. veterans as well [9]. In a recent evaluation of mental health services in the VHA, Watkins and colleagues [10] also found that the average cost of a veteran with a psychiatric disorder was 2.7 times higher than non-psychiatric cases ($12,337/year) and that PTSD was the condition most commonly associated with service utilization (comprising 43% of psychiatric cases in the VHA).

Meta-analytic reviews have unfortunately documented that psychological treatments for PTSD are less effective for improving symptomatology among U.S. veterans than other trauma populations [11], [12]. The Institute of Medicine (IOM) [13] has also raised questions about treating at risk subpopulations of veterans and concluded that “research on treatment of PTSD in U.S. veterans is inadequate to answer questions about interventions, settings, and lengths of treatment that are applicable in this specific population.” Although the VHA has taken steps to enhance the quality of available psychological treatments for PTSD since IOM's review [14]–[16], a recent meta-analytic review of 24 outcome studies for combat-related PTSD [17] documented that these treatments often generated smaller reductions in symptomatology than is customarily expected with professional therapeutic interventions (overall *d* = .49). Goodson and colleagues also documented that residential treatments in particular generated small reductions in PTSD symptomatology (overall *d* = .19), which aligns with earlier results for several smaller studies of individual programs as well [18]–[21]. This outcome literature therefore suggests that while there are promising psychotherapies for combat-related PTSD, the subpopulation of veterans who have the strongest need for effective mental health care are also the most improbable of benefitting from services.

A number of factors may contribute to variability in veterans' responses to psychological treatments for PTSD. When considering the challenges in residential treatments in particular, these programs target severe cases of PTSD in which outpatient options have typically been exhausted. With the wars in Iraq/Afghanistan, residential programs are increasingly treating younger veterans with acute forms of symptomatology [22]. However, when compared to older counterparts from Vietnam and other eras, these veterans may not have experienced the erosion of adaptive resources that often accompanies a longer-term course of PTSD [23]. War-zone service can also entail varying rates of exposure to potential traumas that may differentially increase the risk for PTSD [24] and engender possible moral/ethical challenges related to combat-related decisions/actions [25]. Veterans can also incur physical injuries in the war-zone and/or PTSD symptomatology might reduce physical health status and increase the risk for a variety of medical problems [26], [27]. Of the 25% percent of Iraq/Afghanistan veterans who received a psychiatric diagnosis in the VHA prior to 2005, Seal and colleagues [28] also found that 56% suffered from multiple mental health conditions that may demand clinical attention. Other research has also documented high rates of substance-related problems with combat-exposed samples [29], [30], which represents another significant concern for this population.

These issues with physical and/or psychiatric comorbidity may complicate treatment in many cases of combat-related PTSD [31]–[33]. Studies have documented that clinicians frequently perceive patients with PTSD and co-occurring substance use disorders (SUDs) as being more difficult to treat than either disorder alone [32], [33]. For example, clinicians might struggle with anger and frustration at patients' self-destructive behaviors and lack of insight/judgment into their substance misuse. Notwithstanding recent innovations in psychotherapies for combat-related PTSD [14]–[16], clinicians might also struggle to know how to best prioritize and implement specific evidence-based interventions for PTSD and SUD symptomatology (e.g., beginning exposure prior to sustained period of sobriety). In cases of medical comorbidities, clinicians might similarly struggle to collaborate with other health care professionals outside of their training background and balance the demands of psychological and medical treatments that veterans might require. For example, although research is still limited as to the consequences of physical health status on outcomes of PTSD treatment, veterans' posttraumatic symptomatology might hinder their ability to attend their appointments and adhere to recommendations of medical providers. Similarly, uncontrolled medical problems may conceivably exacerbate posttraumatic symptomatology and limit veterans' energy and motivation to adequately engage in their mental health treatments.

### Study Aims

The VHA will continue to assume primary responsibility for addressing the many forms of PTSD in U.S. veterans. However, in light of increasing demands for services since the U.S. involvement in Iraq/Afghanistan, it is essential to better optimize PTSD residential programs and other high value treatments for combat-related PTSD. Focusing on clinical information from a PTSD residential program over an eight-year period, the overarching purpose of this study was to identify pre-treatment characteristics that are associated with favorable responses to treatment. Namely, we utilized latent class growth analysis (LCGA) to determine trajectories of changes in PTSD symptomatology from baseline through a four-month follow-up assessment. LCGA allows for the empirical examination of the underlying heterogeneity within the data, which is often simply modeled as error in other statistical procedures. Specifically, LCGA tests whether the population under study is composed of a mixture of distinct distributions or “classes” of individuals with differing trajectories of change over time. LCGA also permits modeling of covariates as predictors of class membership. Drawing on LCGA in this study, we anticipated finding variability in veterans' trajectories of PTSD. Namely, when considering past research [11], [12], [17]–[21], we anticipated finding that many of the patients would obtain significant benefit from treatment along with a substantive minority who would not display improvements in PTSD. We also hypothesized that responders would be distinguished from these non-responders by several key clinical concerns for this population – severity of baseline PTSD symptomatology, chronicity of PTSD (i.e., older in age), greater combat exposure, poorer physical health and mental health status, and more problems with substance abuse.

## Method

### Setting and Participants

The present study utilized clinical information for 805 veterans who completed a sixty- to a ninety-day residential PTSD treatment program between 2000 and 2007 at a large medical center in the VHA. This site houses two PTSD Residential Rehabilitation Programs (PRRP), consisting of a 45-bed program for men and 10-bed program for women. The men's program has existed since 1978 and the women's program began nearly twenty years ago. These programs provide treatment to veterans from all eras of military service with combat-related PTSD and related problems. The number of PRRPs in the VHA has varied since their inception in the 1970s and treatment procedures might differ from one program to another. Of the 22 PRRP sites in the VHA at this time, these two have a national catchment area and represent large programs for the men and women whom they serve. Veterans reside in a therapeutic milieu setting during each of these programs in which they participate in a range of psychological interventions throughout the day and evening hours (e.g., discussing traumas via exposure sessions, anger management, stress reduction, communication skills, psychoeducation, interpersonal process groups, parenting skills, recreation therapy). Treatment is exclusively provided in a group format in these programs and largely adheres to a cognitive behavioral framework.

Admissions to these two programs were based on clinician referrals for veterans with severe PTSD symptomatology who had not improved sufficiently through less intensive treatment options. Exclusion criteria included active psychotic symptoms, alcohol/drug misuse within the previous 14 days, and the presence of medical conditions that would significantly interfere with/or prevent their engagement in any treatment activities/procedures. All of these participants had a primary diagnosis of PTSD from the program staff. Although we could not closely monitor diagnostic procedures across these assessments, PTSD diagnoses were based on clinical interviews and objective instruments that are commonly implemented in the VHA (e.g., Posttraumatic Clinical Checklist [PCL]). Diagnostic information for other psychiatric disorders was not available for most of the patients in the sample. In cases where veterans had more than one admission to these programs, we only incorporated information from their first admission in the statistical analyses.

The average length of stay in the two programs at this site was 66 days and the average age in the sample was 51.53 years (*SD*  = 8.03). The sample was predominantly comprised of men (89.1%) and persons who self-identified as Caucasian (59.5%) in their ethnic background. Other ethnicities included African American (16.6%), Latino/a (14.7%), Asian American (2.2%), Native American (1.9%), and other minority groups (5.1%). Nearly half of the veterans were divorced (35.8%) or separated (8.0%), 32.1% were married or living with a domestic partner, 18.4% had never married, and 5.7% had been widowed. On average, these participants had 11.61 years (*SD* = 1.31) of formal education. The median annual income ranged from $20,000 to $30,000. The sample largely included Vietnam Veterans; 4.2% had served in Iraq and/or Afghanistan.

### Procedures

All measures that form the basis of this study were completed primarily for clinical decision-making and quality management of the two residential programs. However, prior to the collection of data, a consent process was approved by Stanford University's Institutional Review Board (IRB) for Human Subjects in Medical Research (Protocol #80713) and the VA Research and Development (R & D) Committee that allowed these residential patients to provide written permission on the pre-treatment questionnaire for their clinical assessments to be used for research purposes. In 2007, this protocol was closed and a de-identified data set was approved by the Stanford IRB and the R & D Committee for the types of research analyses that were conducted in this study (Protocol #12236).

Assessment of PTSD symptom severity was completed via the PCL at pre-treatment, post-treatment, and follow-up (four months after discharge). In addition, veterans completed several other self-report instruments at pre-treatment that might also affect their responses to treatment. Given the aims of the present study, we excluded 221 veterans who were admitted to the program during the same period but only provided information on PTSD at one time point and/or did not complete these other study measures at pre-treatment. When compared to the 805 veterans who are the focus of this study, preliminary analyses revealed that these individuals with an incomplete response reported less combat exposure, *p* = .004. However, these groups did not differ in their PTSD symptom severity at pre-treatment or on any of the remaining variables that form the basis for this study.

### Measures

The Combat Experiences Scale (CES) [34] was used to assess exposure to life-threatening activities/circumstances that may occur in a war-zone (e.g., taking incoming fire, firing weapon, danger of injury/death). The CES is a well-established measure that includes seven items scored on a five-point scale, with anchor points from 1 (*Never*) to 5 (*51+ times*).

The Medical Outcomes Study Short Form (SF-12) [35] was incorporated to assess patients' physical (e.g., mobility, lack of pain, activity restriction) and mental (e.g., feeling calm/peaceful, depressed mood) health. Higher scores indicate better health status in each domain. Other studies have also found that the physical health component of the SF-12 is associated with medical issues [36] and the mental health component is predictive of psychiatric comorbidity (e.g., depression) [37].

Pre-treatment substance abuse was gauged with an 18-item instrument assessing standard problems that can result from heavy alcohol/drug misuse over a number of domains (e.g., legal, financial, residential, interpersonal) [38]. These items were rated on a five-point scale such that higher scores indicated more substance-related problems (1 =  *Never*, 5 =  *Often*).

PTSD symptomatology related to military experiences was assessed at the three time points with the Posttraumatic Stress Disorder Checklist – Military version (PCL-M) [39], [40]. The PCL-M is another widely used self-report instrument assessing distress associated with the 17 symptoms of PTSD in DSM-IV over the past month. Items were rated on a five-point scale, with anchor points of 1 (*Not at all*) to 5 (*Extremely*). A cutoff score of 50 has been recommended for a probable PTSD diagnosis.

### Plan of Analysis

We first calculated descriptive statistics and bivariate correlations between the study variables. Using MPlus Version 6.1 [41], we next performed LCGA to identify groups of veterans (i.e., classes) with unique trajectories of PTSD symptomatology from pre-treatment to post-treatment to follow-up. In light of prior research with LCGA with clinical [42] and non-clinical [43] groups that found longitudinal changes in PTSD symptomatology, we anticipated to also find multiple trajectories of treatment response in this sample. As such, we examined a 1-, 2-, 3-, and 4-class solution, and a variety of fit indices were considered when determining the best-fitting model.

In particular, we considered the Bayesian Information Criterion (BIC) and Akaike Information Criterion (AIC), with lower values indicating better fit, as well as the Vuong-Lo-Mendell-Rubin Likelihood Ratio Test (VLMR-LRT), which tests the relative merits of a given model against a model with one fewer classes (e.g., a 3-class vs. a 2-class model). The VLMR-LRT produces a p value, which represents the probability that a more complex model fits the data just as well as a simpler model with one fewer classes. Thus, a VLMR-LRT with a p<.05 indicates that adding another class to the model significantly improves fit. Simulation research has found that the VLMR-LRT is a particularly promising index in determining the appropriate number of classes [44]. We also report values for entropy, which is a measure of classification uncertainty. Although there is no clear cutoff for acceptable levels of entropy [45], values closer to 1 indicate clear delineation of classes and values closer to 0 indicate greater classification uncertainty [46]. Parameters in the LCGA were estimated using a maximum likelihood robust (MLR) procedure, which is robust even in the presence of non-normal data. Shapiro-Wilk tests revealed that PCL scores at all three time points significantly deviated from normal (all p's<.001).

Several covariates were included in the model based on previous research: age, sex (0 =  women, 1 =  men), ethnicity (0 =  ethnic minority, 1 =  Caucasian), combat exposure, physical health, mental health, and substance abuse. Multinomial logistic regression was used to determine which covariates significantly predicted class membership. Missing data was handled using multiple imputation, which has the advantage of providing unbiased estimates while making use of all available data. Five imputations were performed, which is “sufficient to obtain excellent results” (p. 548) [47]. Only 2 participants had a missing PCL score at baseline; 143 had a missing PCL score at post-treatment; and 326 had a missing PCL score at follow-up. In order to be included in the present study, a participant could not omit more than 3 items on the PCL and was required to have valid PCL scores at a minimum of two assessments.

## Results

### Bivariate Analyses

Descriptive statistics and bivariate correlations are outlined in Table 1. All of the study variables were linked with PTSD symptom severity at least at one of the assessment points. Combat exposure, baseline health status, and problems with substance abuse were correlated with veterans' symptomatology at each of the three assessments in the anticipated directions.

**Table 1:** Descriptive Statistics and Bivariate Correlations Between Study Variables.

	*M*	*SD*	PTSD at Pre-treatment	PTSD at Post-treatment	PTSD at Follow-up
**Age**	51.632	8.066	−.085*	.024	.055
**Sex**	0.891	0.311	.065	.153***	.123***
**Ethnicity**	0.595	0.491	−.072*	−.050	−.012
**Combat Exposure**	21.924	12.272	.158***	.224***	.127***
**Physical Health Status**	38.809	10.138	−.169***	−.136***	−.126***
**Mental Health Status**	30.760	9.083	−.336***	−.224***	−.197***
**Substance Abuse Problems**	32.950	17.690	.218***	.134***	.119***

**Note** : ***p<.001, **p<.01, *p<.05; *M*  =  mean, *SD*  =  standard deviation, PTSD  =  Posttraumatic Stress Disorder. Sex was coded in such a manner that 0 =  Women, 1 =  Men. Ethnicity was coded such that 0 =  Non-Caucasian, 1 =  Caucasian persons.

### Latent Class Growth Analysis Findings

**Figure 1:**
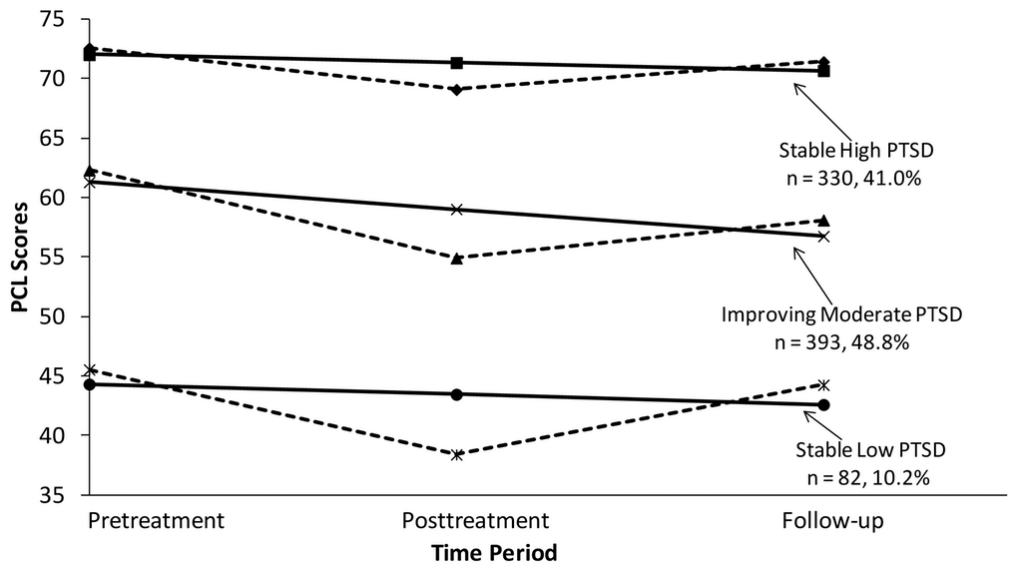
Graphical depiction of the 3-class solution for PCL-M scores from pre-treatment to a four-month follow-up assessment. Solid lines represent trajectories based on estimated means. Dotted lines represent trajectories based on actual means in the sample.

After testing a 1-, 2-, 3-, and 4-class model in the LCGA, a 3-class model was clearly found to provide the best fit to the data (see Table 2). A graphical depiction of this model is provided in Figure 1. In this figure, solid lines represent estimated means based on the LCGA probabilistic model. These estimated trajectories are straight lines since we had three time points and could only test linear models (i.e., a quadratic model would require 4 or more longitudinal observations). Dotted lines in this figure represent trajectories based on actual sample means and therefore deviate from the probabilistic LCGA model and do not conform to a perfect linear pattern.

**Table 2:** Goodness of Fit Indices for Latent Class Growth Analysis Examining Posttraumatic Stress Symptoms from Pretreatment to Follow-up (N = 805).

	AIC	BIC	Entropy	VLMR-LRT *p* value
**1-Class**	19124.116	19147.570	—	—
**2-Class**	18533.766	18604.128	.732	<.001
**3-Class**	18350.847	18468.118	.735	<.001
**4-Class**	18339.694	18503.874	.785	.114

**Note**: AIC  =  Akaike Information Criterion, BIC  =  Bayesian Information Criterion, VLMR-LRT  =  Vuong-Lo-Mendell-Rubin Likelihood Ratio Test. Missing data was handled using multiple imputation with five imputed data sets. Mean values across these data sets are presented for AIC, BIC, and Entropy, and the median p value is presented for the VLMR-LRT.

As shown in Figure 1, 48.8% exhibited a trajectory characterized by moderate levels of PTSD symptomatology that significantly declined from pre-treatment to follow-up (Intercept  = 61.322, *p*<.001; Slope  = −2.274, *p*<.001). Veterans with this trajectory were considered to have “Improving Moderate PTSD.” A smaller subset of individuals (41.0%), labeled the “Stable High PTSD” group, showed more severe baseline levels of PTSD symptomatology that remained fairly stable (Intercept  = 72.043, *p*<.001; Slope  = −0.672, *p* = .158). A third group (10.2%) displayed relatively low initial levels of PTSD symptoms that also remained relatively stable over time (Intercept  = 44.322, *p*<.001; Slope  = −0.859, *p* = .523); individuals with this trajectory made up a “Stable Low PTSD” group. To ensure that these results were not biased due to our method of handling missing data, these analyses were repeated using the method of listwise deletion. These analyses also supported a three-class model with highly similar frequencies of participants within each class (i.e., 48.7%, 43.0%, and 8.4% in the moderate, high, and low PTSD groups, respectively).

Given the observed discrepancy between some of the estimated means from the LCGA model and the actual means in the sample (particularly at post-treatment), mean PCL scores were compared within each class at all three assessments as well as effect sizes for the three groups for changes in PTSD symptomatology from pre- to post-treatment, pre-treatment to follow-up, and post-treatment to follow-up. As shown in Table 3, each group reported significant reductions in PTSD symptomatology from pre- to post-treatment (*d*s = −.42 to −.78). However, all three groups reported significant increases in symptomatology from post-treatment to follow-up (*d*s  = .26 to .59), indicating some degree of relapse in the months after treatment. When compared to their PCL scores at baseline, only those in the Improving Moderate PTSD group showed reductions in symptomatology that remained significantly different from zero at follow-up (*d*  = −.50).

**Table 3:** Comparison of Mean PTSD Scores at Pre-treatment, Post-treatment, and Follow-up for the 3-Class Model (N = 805).

	Mean: Pre-treatment (Pre)	Mean: Post-treatment (Post)	Mean: Follow-up	Wald Test: Pre vs. Post	Pre to Post *d*	Wald Test: Pre- vs. Follow-up	Pre to Follow-up *d*	Wald Test: Post vs. Follow-up	Post to Follow-up *d*
**Stable High PTSD (n = 330)**	72.594	69.107	71.442	26.971**	−0.42	2.832	−0.15	8.362*	0.26
**Improving Moderate PTSD (n = 393)**	62.324	54.931	58.127	96.716**	−0.78	31.910**	−0.50	17.136**	0.32
**Stable Low PTSD (n = 82)**	45.554	38.404	44.262	32.497**	−0.77	0.934	−0.13	12.021**	0.59

**Note**: PTSD  =  Posttraumatic Stress Disorder. **p<.001, *p<.01.

Examination of the covariates in the LCGA model revealed that the Stable High PTSD group was primarily characterized by greater combat exposure and poorer status in both physical and mental health compared to the Improving Moderate PTSD group (see Table 4). In contrast, those in the Stable Low PTSD group were older, experienced fewer combat stressors, reported better physical and mental health status, and had fewer problems with substance abuse compared to the Improving Moderate PTSD group. When the Stable Low PTSD and Stable High PTSD groups were compared directly, the Stable High PTSD group was found to have greater combat exposure, poorer mental and physical health, and more problems with alcohol.

**Table 4:** Multinomial Logistic Regression Analysis Predicting Class Membership for the 3-Class Model (N = 805).

	Stable High PTSD vs. Improving Moderate PTSD				Stable Low PTSD vs. Improving Moderate PTSD				Stable High PTSD vs. Stable Low PTSD			
	B	SE	Odds Ratio	p	B	SE	Odds Ratio	p	B	SE	Odds Ratio	p
**Age**	0.006	0.014	1.006	.676	0.051	0.025	1.052	.045	−0.045	0.026	0.956	.081
**Sex**	0.200	0.478	1.221	.677	0.080	0.554	1.083	.885	0.120	0.685	1.127	.862
**Ethnicity**	−0.295	0.233	0.744	.205	0.361	0.364	1.435	.321	−0.656	0.379	0.519	.083
**Combat Exposure**	0.022	0.011	1.022	.055	−0.053	0.020	0.948	.007	0.075	0.022	1.078	.001
**Physical Health Status**	−0.046	0.014	0.955	.001	0.056	0.018	1.057	.002	−0.102	0.021	0.903	<.001
**Mental Health Status**	−0.080	0.018	0.923	<.001	0.089	0.020	1.093	<.001	−0.169	0.025	0.844	<.001
**Substance Abuse**	0.011	0.007	1.011	.154	−0.033	0.012	0.967	.006	0.044	0.013	1.045	<.001

**Note**: PTSD  =  Posttraumatic Stress Disorder.

## Discussion

This study revealed three distinct trajectories of changes in PTSD symptomatology. As anticipated, nearly half of the patients (48.8%) demonstrated significant reductions in PTSD at post-treatment that were maintained at follow-up. The next most common trajectory entailed a Stable High PTSD group (41%) and another smaller minority (10.2%) of patients consistently hovered below the cutoff for symptom severity on the PCL. Although veterans in these groups had significant reductions in PTSD symptom severity at discharge, neither group generated significant therapeutic benefits at follow-up. Of note, we also did not find evidence for a class of patients who worsened during and/or after their admission. However, in keeping with earlier outcomes with smaller samples of residential patients [17]–[21], a shallow v-shaped pattern of changes in PTSD was observed across these three trajectories. Namely, all veterans reported a significant amelioration of PTSD at discharge but then went on to experience a resurgence of symptomatology in the four months after the program. When considering the Stable High and Low PTSD trajectories in particular, these relapses unfortunately negated gains from treatment. Although we lacked information on factors that contributed to these relapses, previous research suggests that limited psychosocial resources and possible environmental deficiencies outside of the program serve as important maintenance factors for PTSD among many veterans with this condition [23].

Other results indicated that veterans who responded favorably to treatment could be distinguished by several pre-treatment factors. When considering PTSD symptom severity, veterans in the Moderate Improving PTSD group scored in an intermediate range relative to those who returned to their baseline levels of symptomatology. In addition, patients with an improving trajectory had superior physical and mental health status than those in the Stable High PTSD group. In contrast, when compared to the Stable Low PTSD group, veterans with an improving trajectory indicated poorer health status before treatment began. Results from this second comparison also revealed that these responders were generally younger and had more problems with substance abuse than the Stable Low PTSD group, each of which conflicted somewhat with our hypotheses. This pattern rather suggests that PTSD treatment could have the greatest probability of success in an intermediate range of symptom severity and co-occurring medical/psychiatric problems. From a clinical standpoint, the ideal patient might therefore need to reach a certain level of distress and/or impairment to garner the necessary motivation to engage in therapeutic activities/procedures. However, in cases of severe PTSD in which patients might also find themselves struggling with serious comorbid medical/psychiatric issues, clinicians could encounter greater challenges in addressing trauma-related concerns in an effective manner.

This same pattern for treatment outcomes was also observed for exposure to combat. Namely, although bivariate correlations supported a well-established association between combat exposure and severity of posttraumatic symptomatology [48], patients were in fact the most likely to benefit from treatment if they encountered a certain degree of potentially traumatic scenarios during their war-zone service. However, once these patients had surpassed a possible threshold of exposure, results indicated that the possibility of responding favorably to treatment was lessened. Other research has documented that under conditions of severe exposure to the types of life-threatening stressors assessed in this study, veterans will be more likely to encounter a diversity of additional traumas that may create unique challenges for treating combat-related PTSD [24], [25]. Hence, when focusing on the Stable High PTSD group in particular, many of these patients might have been struggling to resolve other salient traumas (e.g., killing, atrocities) that were not captured by the traditional combat exposure measure used in this study. Several researchers have piloted interventions that may address shame/guilt and other dimensions of moral injury in evidence-based treatments for combat-related PTSD [49], [50].

It was notable that veterans' ages and problems with alcohol/drugs did not significantly differ for the Moderate Improving PTSD and Stable High PTSD groups. These results could be attributable to a restricted range in these variables in that the program was just beginning to serve Iraq/Afghanistan veterans when the data was collected and substance-related problems are quite common in these residential contexts. However, these results also raise questions about whether or not chronicity of PTSD and problems with substance misuse represent barriers for treatment with this population to the same degree as medical issues and psychiatric comorbidities. Recent results from two samples of VHA PTSD residential patients unexpectedly found that those with comorbid SUDs had superior PTSD outcomes compared to cases of PTSD only [51]. Findings such as these highlight the potentially synergistic effects of PTSD and SUDs in the therapeutic context. For example, as SUD symptoms will be confronted via a period of forced sobriety in residential treatment, veterans might be positioned to emotionally process their traumas (and vice versa). VHA clinicians are frequently well-versed in integrative methods with demonstrated efficacy for addressing PTSD and SUDs [52]. The present results additionally support the need for integrated health care and close collaboration between mental health clinicians and medical providers in the care of veterans with combat-related PTSD.

### Limitations

Any conclusions drawn from this study should be tempered against several limitations. We already noted the predominance of veterans from the Vietnam era. With the expanding role of women in the military over recent decades, there was similarly a high predominance of men in this group. As such, these results may not generalize as well to women and the new generation of Iraq/Afghanistan veterans, and future studies on PTSD treatment responses will do well to over-sample these subgroups. With our exclusive focus on veterans presenting for residential treatment at a single site, these results may also not apply to non-clinical populations of veterans, those with sub-threshold PTSD, or veterans who pursue treatment at other PRRPs in the VHA. It was notable that patients in the Stable Low PTSD group generally did not exceed the threshold on the PCL [31]. However, this trajectory occurred with the least frequency and the LCGA results might not generalize to outcomes with less severe cases that clinicians may treat in other settings. For instance, considering the greater representation of older veterans with lower combat exposure in the Stable Low PTSD group, it is possible that some of these persons might not have identified as easily with other veterans in their cohorts and/or many of them could have sought treatment for different reasons. Some of these patients might have accordingly benefitted from a more individualized approach or greater homogeneity in their cohort. These are constant tensions with treating combat-related PTSD in residential settings that we did not have information to address in our study [15].

Given the multiple interventions that veterans received in their treatment, we also could not examine the relations between specific components of the residential programs with the PTSD trajectories derived in the LCGA. Research has demonstrated that the VHA's systematic implementation of evidence-based, trauma-focused psychotherapies for PTSD (e.g., cognitive processing therapy, prolonged exposure) has increased the efficacy of treatments in both residential [14] and outpatient [16] settings. However, because of changes in program staff and introduction of these interventions over the study period, the treatment programs would not have been exactly the same for each of the patients in this sample. As such, we lacked information for testing the incremental benefit of specific components of the overall treatment. Although this clinical sample provided a unique opportunity to address our study aims, future research should examine the helpfulness of different treatment components in a more systematic manner. The assessment of combat exposure and PTSD was also restricted to life-threatening stressors and DSM-IV's emphasis on fear-based symptomatology. In keeping with recent changes in DSM 5 criteria for PTSD, there is increasing consensus that combat-related PTSD can emerge from a much more diverse set of war-zone experiences that frequently entail a far wider realm of emotions than many clinicians and researchers have historically appreciated [25]. As such, we possibly missed important factors for distinguishing patients who displayed improvement in PTSD vs. the remainder of the sample who did not achieve lasting reductions in their trauma-related symptomatology.

### Conclusion

Notwithstanding these limitations, this study addresses a pressing public health concern and notable strengths include the relatively large sample size, focus on a PTSD residential sample, and sophisticated statistical methodology. Findings generally align with other research that documented a lack of positive effects for PTSD residential programs in alleviating the symptomatology of U.S. veterans [17]–[21]. However, the present study also identified several patient characteristics that were prospectively associated with varying trajectories in PTSD outcomes that clinicians might consider in their work. When compared to non-responders (follow-up *d*s  = −.15 and −.13), patients who had responded favorably to treatment (follow-up *d* = −.50) scored in an intermediate range in symptom severity, physical/mental health status, and combat exposure. This pattern supports the clinical wisdom that the ideal patient for PTSD treatment will neither be too healthy or debilitated by psychiatric and medical problems. In the former situation, clinicians may explore other less expensive treatment options for addressing veterans' concerns. However, when considering the 41% of this sample who maintained high levels of PTSD despite the completion of an intensive residential program, this study reinforces the need for more effective strategies for helping this extreme subpopulation of veterans seeking services for PTSD in the VHA. The present results suggest that clinicians should be prepared to assume a synergistic and holistic approach in these cases, which may combine evidence-based treatments for alleviating PTSD symptomatology with other strategies for promoting veterans' health in physical, social, and potentially spiritual domains. With a new generation of veterans now relying on services in an already burdened VHA system, it will be critical for providers from multiple disciplines to collaborate together in optimizing the treatment of combat-related PTSD in the years to come.
